# A Mistake

**Published:** 1868-01

**Authors:** 


*A Mistake.*—We quote the following from the Phila. Dental Quarterly: —"R. D. C., of Stockton, California.—Your tickets for a course of instruction at our Philadelphia Dental Schools, will cost you about $135. You can get board for from $5 to $8 per week. In Baltimore it is more costly. We decline to direct you to any particular college."

The tickets for a course of instruction in the Baltimore Dental College will cost $125, including anatomical dissections. Board can be had in Baltimore at $4.50 to $7 per week.

